# Supplementary figures and images for: Dietary change without caloric restriction maintains a youthful profile in ageing yeast
Source: PLoS Biol. 2023 Aug 29;21(8):e3002245. doi: 10.1371/journal.pbio.3002245 (PMC10464975; doi:10.1371/journal.pbio.3002245)

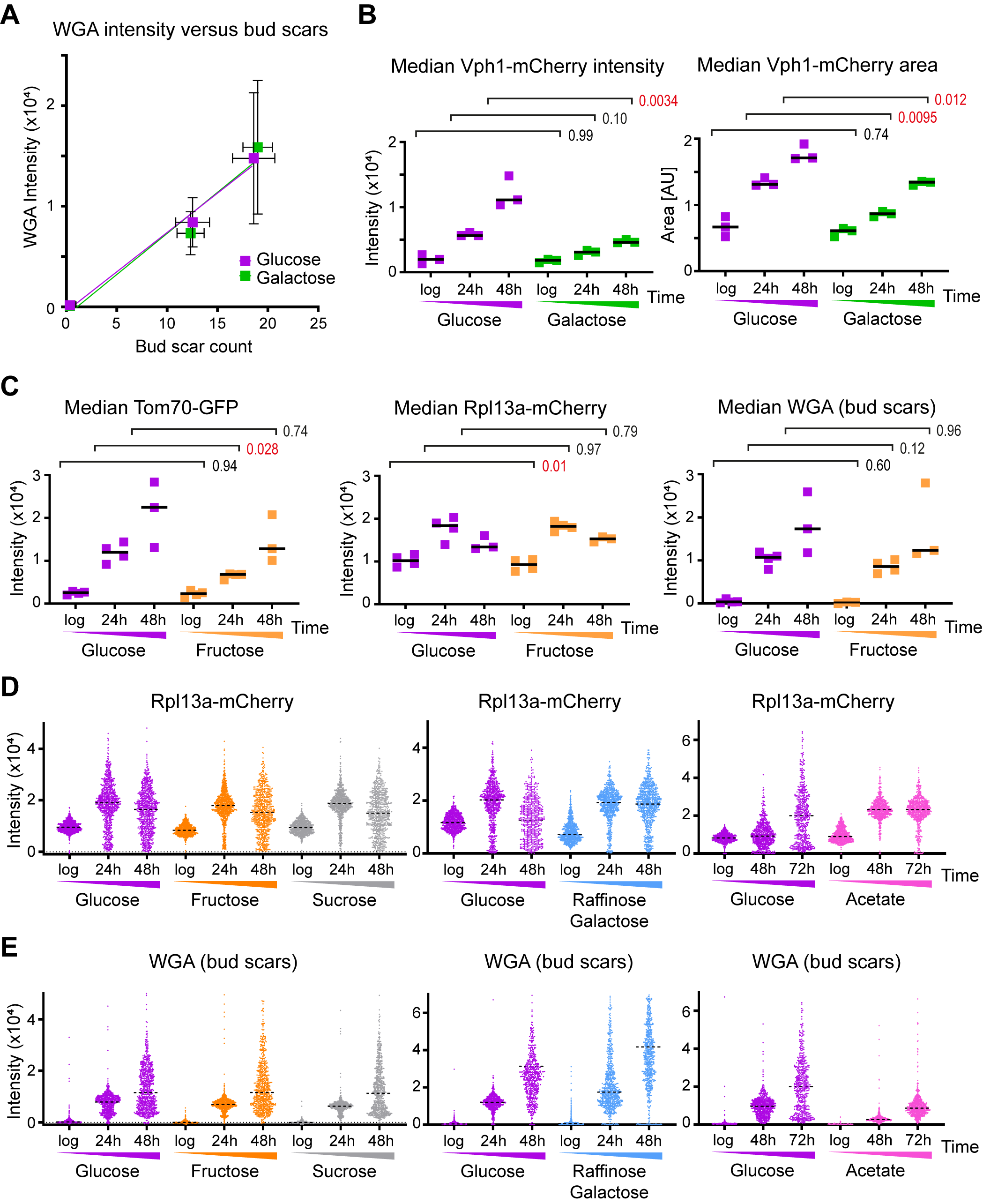

Supplement: S1 Fig — (A) WGA intensities versus manual bud scar counts across ageing on glucose and galactose. (B) Median Vph1-mCherry intensities and areas at different ages on glucose and galactose, as Fig 1C, n = 3. (C) Median Tom70-GFP, Rpl13a-mCherry and WGA intensity for populations aged on glucose or fructose, n = 4 (log, 24 h), n = 3 (48 h), analysed as in Fig 1C. Matching Rpl13a-mCherry and WGA data shown in S1C Fig. (D) Rpl13a-mCherry data for samples in Fig 1G. (E) WGA data for samples in Fig 1G. The data underlying this figure can be found in S2 File. (TIF) [file pbio.3002245.s001.tif]

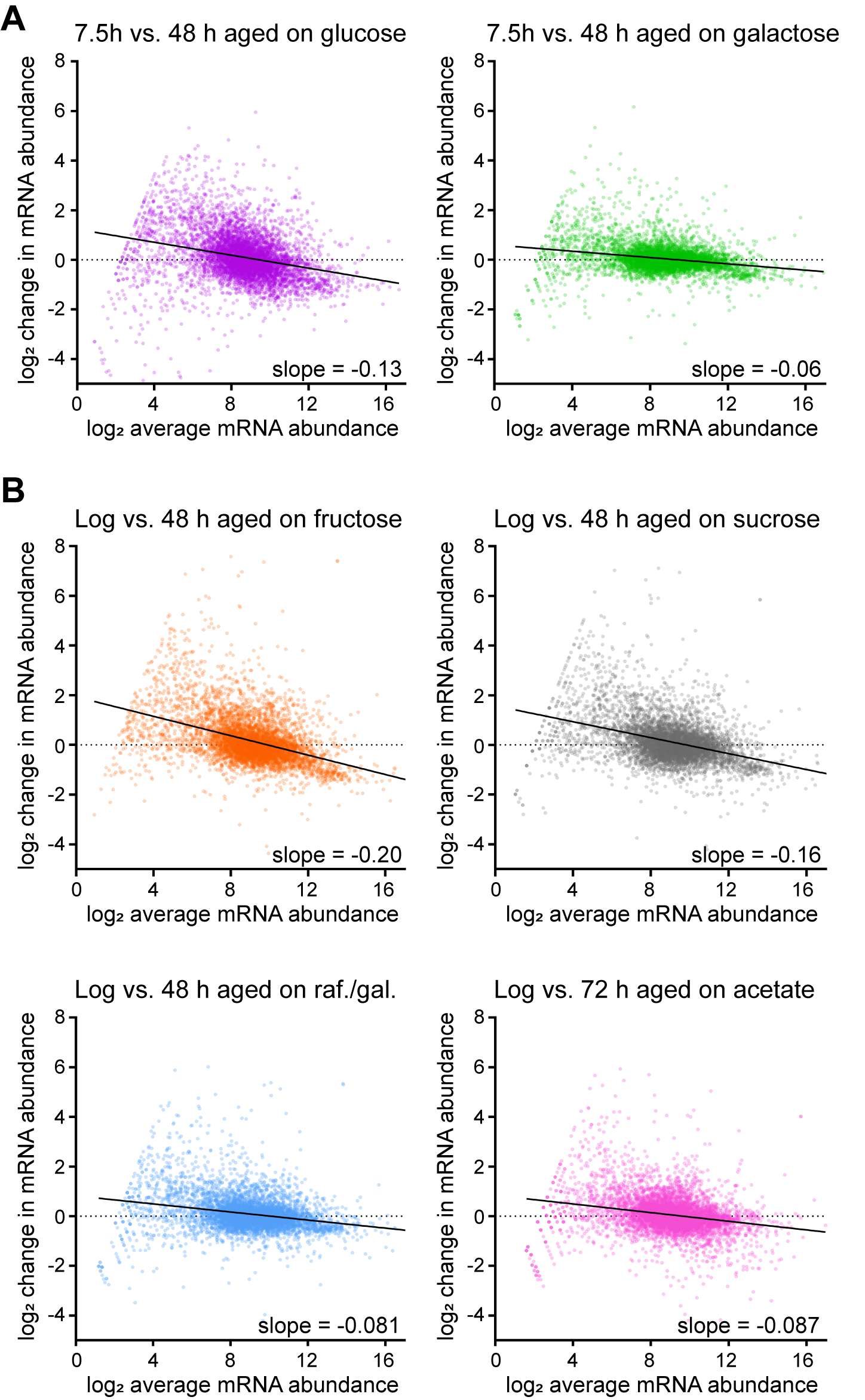

Supplement: S2 Fig — (A) MA plots for gene expression change between 7.5 h and 48 h ageing for wild-type cells on glucose and galactose. Abundances are size-factor normalised log2 transformed read counts from poly(A) selected sequencing libraries, genes with <4 counts at log phase were filtered as changes cannot be accurately quantified for these. Slopes were calculated by linear regression. (B) MA plots for gene expression change between log phase and 48 h ageing for wild-type cells on fructose, sucrose, or a 2% raffinose 0.5% galactose mixture, and equivalent plot for cells aged for 72 h on 2% potassium acetate. Abundances are size-factor normalised log2 transformed read counts from poly(A) selected sequencing libraries, genes with <4 counts at log phase were filtered as changes cannot be accurately quantified for these. Slopes were calculated by linear regression. The data underlying this figure can be found in S2 File. (TIF) [file pbio.3002245.s002.tif]

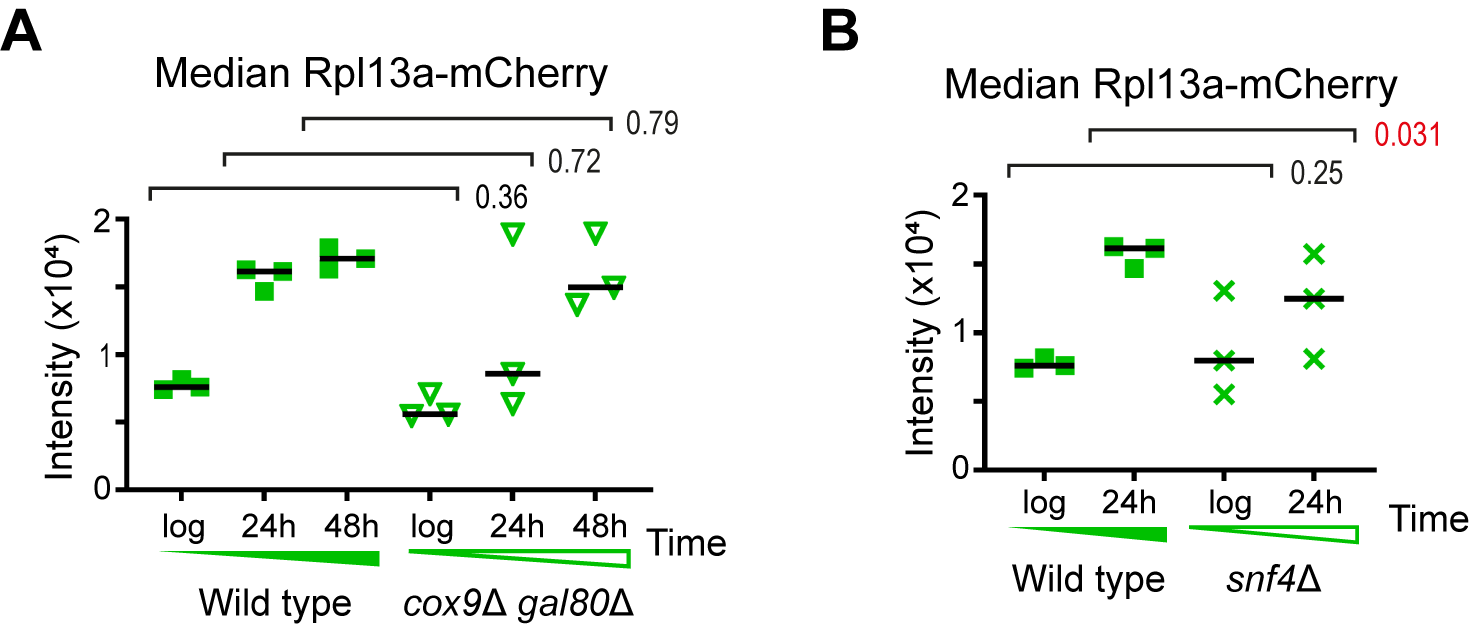

Supplement: S3 Fig — (A) Rpl13a-mCherry data for samples in Fig 3B. (B) Rpl13a-mCherry data for samples in Fig 3D. The data underlying this figure can be found in S2 File. (TIF) [file pbio.3002245.s003.tif]

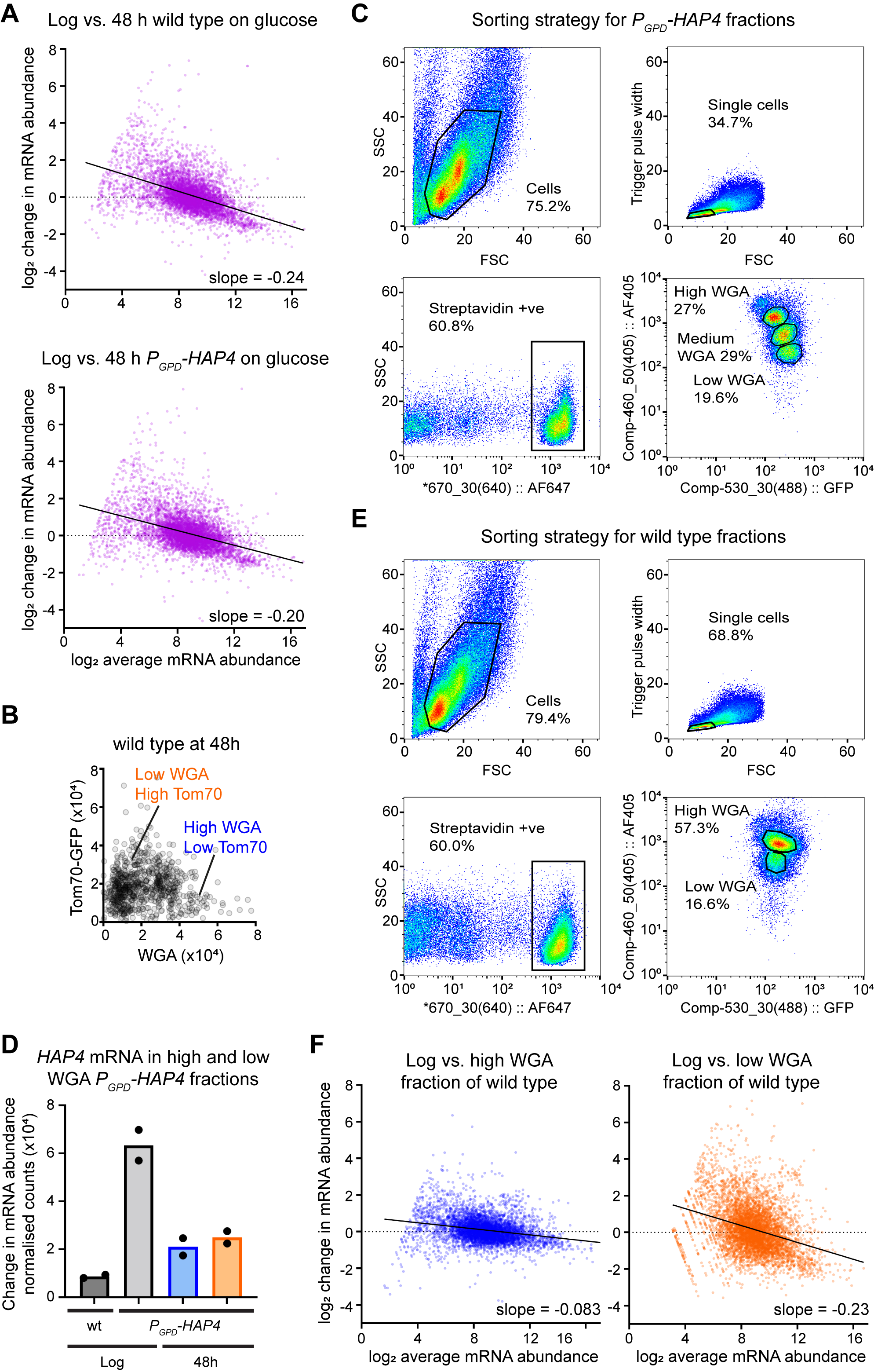

Supplement: S4 Fig — (A) MA plots comparing 48 hour-aged on glucose to log phase control samples for wild type (left) and PGPD-HAP4 (right). Average of 2 biological replicates. (B) Plot of Tom70-GFP versus WGA for wild-type 48 hour-aged population, highlighting subpopulations demarked by high Tom70-GFP, low WGA (orange) and low Tom70-GFP, high WGA (blue). (C) Flow sorting strategy and gating for PGPD-HAP4 cells aged for 48 h on glucose. (D) HAP4 mRNA abundance in PGPD-HAP4 48 hour-aged cells compared to wild-type controls, n = 2 biological replicates. (E) Flow sorting strategy and gating for wild-type cells aged for 48 h on glucose. (F) MA plots showing change in mRNA abundance from log phase to the high WGA (left, blue) or low WGA (right, orange) subpopulations of 48 hour-aged wild-type cells purified by flow cytometry in E. Cut-off for low expressed genes on which quantification is unreliable at 16 normalised counts; n = 1. The data underlying this figure can be found in S2 File. (TIF) [file pbio.3002245.s004.tif]

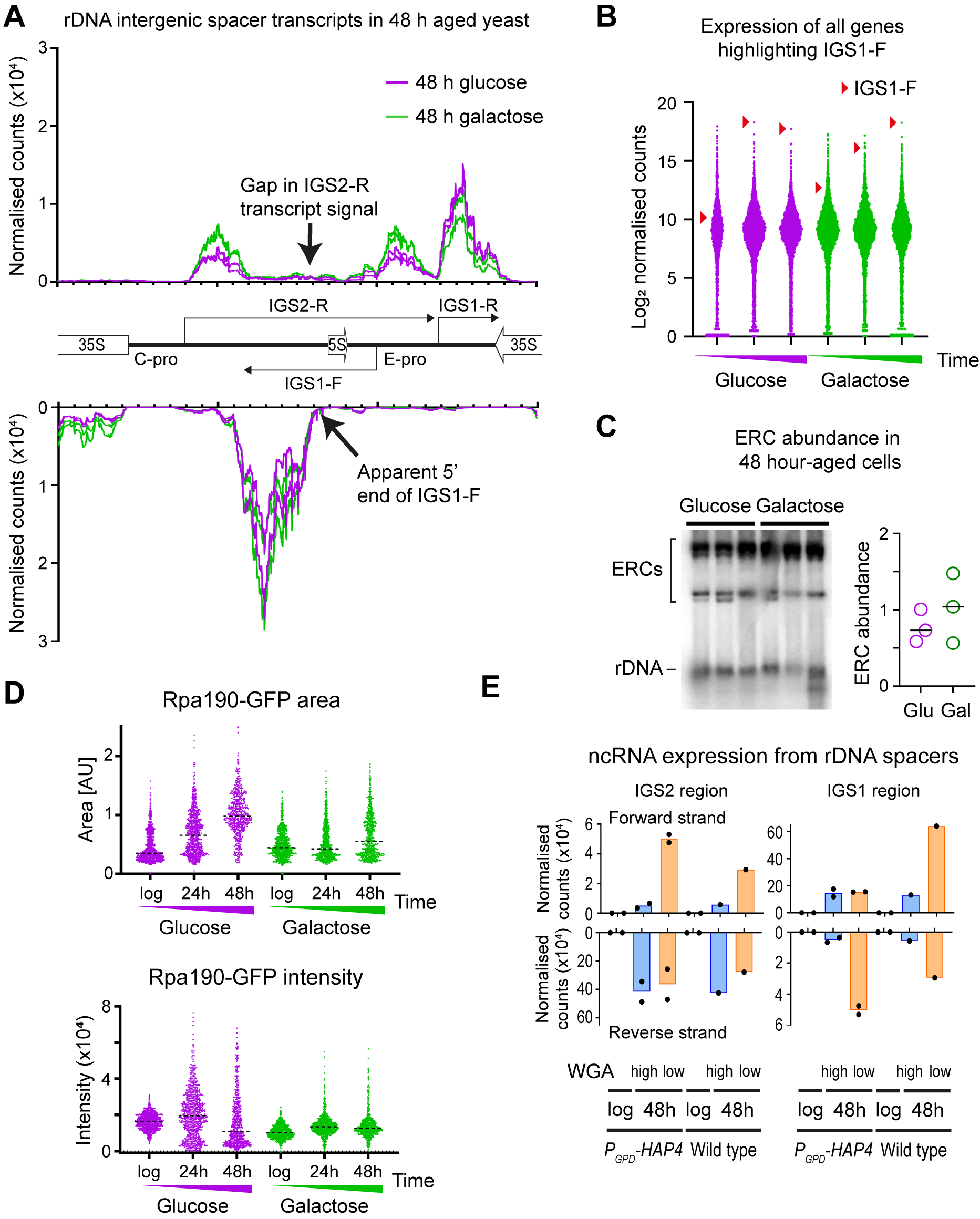

Supplement: S5 Fig — (A) RNA read counts for each strand in 10 bp windows across rDNA intergenic spacer regions, for 2 biological replicate samples each on aged for 48 h on glucose or galactose. Previously described promotors and RNA species are indicated, along with deviations from expected profiles. (B) Log2 normalised read counts for all genes including the rDNA intergenic spaces. The individual dot in each sample corresponding to the rDNA intergenic spacer probe containing the IGS1-F ncRNA is indicated by a red arrow. (C) Southern blot analysis of ERCs and chromosomal rDNA in 3 biological replicates each of wild-type cells aged for 48 h on glucose and galactose. Quantification is of the ratio between ERCs and chromosomal DNA. (D) Distribution of signal areas and intensities from flow cytometry images of Rpa190-GFP in populations of log phase cells or mother cells aged for 24 and 48 h in YP with 2% glucose or galactose. A total of 1,000 cells are imaged per population after gating for circularity (all) and biotin (aged cells only, based on streptavidin-Alexa647). Images are post-filtered for focus of Rpa190-GFP, leaving approximately 600 cells per population. (E) rDNA ncRNA abundances in the sorted fractions of PGPD-HAP4 and wild-type cells from Fig 4. The data underlying this figure can be found in S2 File and S1_Raw_Images.pdf. (TIF) [file pbio.3002245.s005.tif]

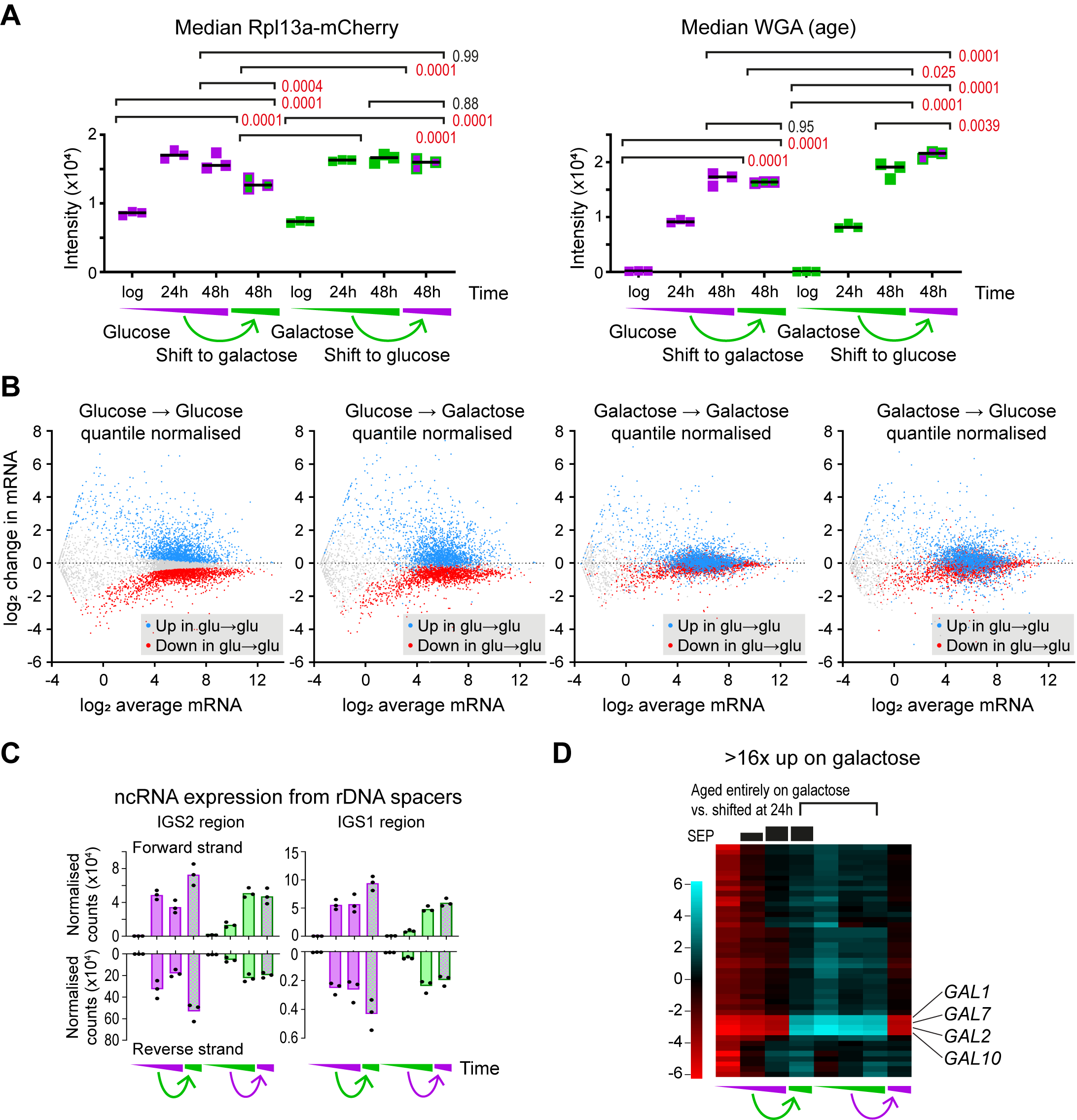

Supplement: S6 Fig — (A) Rpl13a-mCherry and WGA intensity data for samples in Fig 6B. (B) MA plots showing change in abundance between log and 48 h relative to average abundance for each mRNA, highlighting genes that change significantly during ageing in glucose. Abundances are size-factor normalised log2 transformed read counts from poly(A) selected sequencing libraries, datasets were quantile normalised to match overall distributions, and genes with <4 counts at log phase were filtered out. Data is average of 3 biological replicates, significant differential gene expression calculated using DESeq2 with p < 0.01. (C) rDNA ncRNA abundances for media shift samples. (D) Hierarchical clustering across all samples of genes either induced >16× on galactose relative to glucose at log phase, with primary galactose metabolic genes annotated. The extent of SEP entry is indicated diagrammatically above the plots based on Tom70-GFP intensities from B. Average of 3 biological replicates. The data underlying this figure can be found in S2 File. (TIF) [file pbio.3002245.s006.tif]
