# Supplementary material for: Dietary change without caloric restriction maintains a youthful profile in ageing yeast
Source: PLoS Biol. 2023 Aug 29;21(8):e3002245. doi: 10.1371/journal.pbio.3002245 (PMC10464975; doi:10.1371/journal.pbio.3002245)
Supplement: S1 Table — All strains are diploid derivatives of the MEP system [52]. TOM70-GFP, VPH1-mCherry, and RPL13a-mCherry markers are heterozygous to avoid growth defect. (DOCX) [file pbio.3002245.s007.docx]

| DH18 | MEP diploid TOM70-GFP RPL13A-mCherry | *ade2::hisG his3 leu2 met15D::ADE2/MET15 lys2/LYS2 ura3DO trp1D63 hoD::SCW11pr-Cre-EBD78-NatMX loxP-UBC9-loxP-LEU2 loxP-CDC20-Intron-loxP-HPHMX Tom70-GFP-TRP1/+ RPL13A-mCherry-Kan/+* |
| --- | --- | --- |
| DH17 | MEP diploid TOM70-GFP VPH1-mCherry | *ade2::hisG his3 leu2 met15D::ADE2/MET15 lys2/LYS2 ura3DO trp1D63 hoD::SCW11pr-Cre-EBD78-NatMX loxP-UBC9-loxP-LEU2 loxP-CDC20-Intron-loxP-HPHMX Tom70-GFP-TRP1/+ VPH1-mCherry-Kan/+* |
| DH179 | MEP diploid VPH1-mCherry RPA190-GFP | *ade2::hisG his3 leu2 lys2/met15D::ADE2 ura3DO trp1D63 hoD::SCW11pr-Cre-EBD78-NatMX loxP-UBC9-loxP-LEU2 loxP-CDC20-Intron-loxP-HPHMX VPH1-mCherry-Kan RPA190-GFP-HIS3* |
| DH60 | MEP diploid TOM70-GFP RPL13A-mCherry HAP4 overexpression | *ade2::hisG his3 leu2 met15D::ADE2/MET15 lys2/LYS2 ura3DO trp1D63 hoD::SCW11pr-Cre-EBD78-NatMX loxP-UBC9-loxP-LEU2 loxP-CDC20-Intron-loxP-HPHMX Tom70-GFP-TRP1/+ RPL13A-mCherry-Kan/+ KanMX6-Pgpd-HAP4/+* |
| DH126 | MEP diploid TOM70-GFP RPL13A-mCherry snf4Δ | *ade2::hisG his3 leu2 met15D::ADE2/MET15 lys2/LYS2 ura3DO trp1D63 hoD::SCW11pr-Cre-EBD78-NatMX loxP-UBC9-loxP-LEU2 loxP-CDC20-Intron-loxP-HPHMX Tom70-GFP-TRP1/+ RPL13A-mCherry-Kan/+ snf4::URA3* |
| DH197 | MEP diploid TOM70-GFP RPL13A-mCherry cox9Δ gal80Δ | *ade2::hisG his3 leu2 met15D::ADE2/MET15 lys2/LYS2 ura3DO trp1D63 hoD::SCW11pr-Cre-EBD78-NatMX loxP-UBC9-loxP-LEU2 loxP-CDC20-Intron-loxP-HPHMX Tom70-GFP-TRP1/+ RPL13A-mCherry-Kan/+ cox9::URA3 gal80::HIS3* |
