# Supplementary material for: Dietary change without caloric restriction maintains a youthful profile in ageing yeast
Source: PLoS Biol. 2023 Aug 29;21(8):e3002245. doi: 10.1371/journal.pbio.3002245 (PMC10464975; doi:10.1371/journal.pbio.3002245)
Supplement: S2 Table — (DOCX) [file pbio.3002245.s008.docx]

| oJH1520 | TOM70 DN45 1 | TAGTTTTTGTCTTCTCCTAAAAGTTTTTAAGTTTATGTTTACTGT GAATTCGAGCTCGTTTAAAC |
| --- | --- | --- |
| oJH1521 | TOM70 UP45 1 | AAGATTCAAGAAACTTTAGCTAAATTACGCGAACAGGGTTTAATG CGG ATC CCC GGG TTA ATT AAC |
| oDH11 | RPL13A-mCherry UP45 | AAGAGAGCTAGAGAAAAGGCTGAAGCTGAAGCTGAAAAGAAGAAATGCATGCTTATGGTGAGCAA |
| oDH12 | RPL13A-mCherry DN45 | ATACAAAAATTGTGGATGAAAAATTCTTTGATGAAGTTTTTAGATAAGTTATACTAGTTCGTCGACTGGAT |
| oDH1 | VPH1 UP45 1 | GACATGGAAGTCGCTGTTGCTAGTGCAAGCTCT TCC GCT TCA AGC TGC ATG CTT ATG GTG AGC AA |
| oDH2 | VPH1 DN45 1 | AATGAAGTACTTAAATGTTTCGCTTTTTTTAAAAGTCCTCAAAAT AAGTTATACTAGTTCGTCGACTGGAT |
| oDH17 | HAP4 UP45 | CTATCTACAGGTCCACTTTACACTTAATAATATAAAAATACTACTCGTACGCTGCAGGTCGAC |
| oDH18 | HAP4 DN45 | ACGAGGGCGACTAGCGGAGGCCTGTAGTAGAAAAGTCTTTGCGGTCATCGATGAATTCTCTGTCG |
| oDH60 | SNF4 UP45 | TTCTGCTGTGTTAGCATTAGGAGGAAGCGAAAAGGAAAATACATA CGGATCCCCGGGTTAATTAAG |
| oDH61 | SNF4 DN45 | TTGCATTTATTTATAGTATGTACACAAAAATCTCATCGGCTCGTT GAATTCGAGCTCGTTTAAAC |
| oDH113 | COX9 UP45 | AGCAAGATATTTGCAAACTACTAACTACAAGCGACTTACACAGAC CGGATCCCCGGGTTAATTAAG |
| oDH114 | COX9 DN45 | TAGGAATAAGAATATAATGCGAAAAACAATAGTGGTCAGGTTCGG GAATTCGAGCTCGTTTAAAC |
| oDH117 | GAL80 UP45 | GTATACAATCTCGATAGTTGGTTTCCCGTTCTTTCCACTCCCGTC CGGATCCCCGGGTTAATTAAG |
| oDH118 | GAL80 DN45 | TATAACGTTCGCTGCACTGGGGGCCAAGCACAGGGCAAGATGCTT GAATTCGAGCTCGTTTAAAC |
| oAZ114 | RPA190 tag UP45 | GGTACGGGTTCATTTGATGTGTTAGCAAAGGTTCCAAATGCGGCT CGGATCCCCGGGTTAATTAAC |
| oAZ115 | RPA190 tag DN45 | AAACTAATATTAAATCGTAATAATTATGGGACCTTTTGCCTGCTT GAATTCGAGCTCGTTTAAAC |
