# Supplementary material for: Dietary change without caloric restriction maintains a youthful profile in ageing yeast
Source: PLoS Biol. 2023 Aug 29;21(8):e3002245. doi: 10.1371/journal.pbio.3002245 (PMC10464975; doi:10.1371/journal.pbio.3002245)
Supplement: S1 Raw images — (PDF) [file pbio.3002245.s011.pdf]

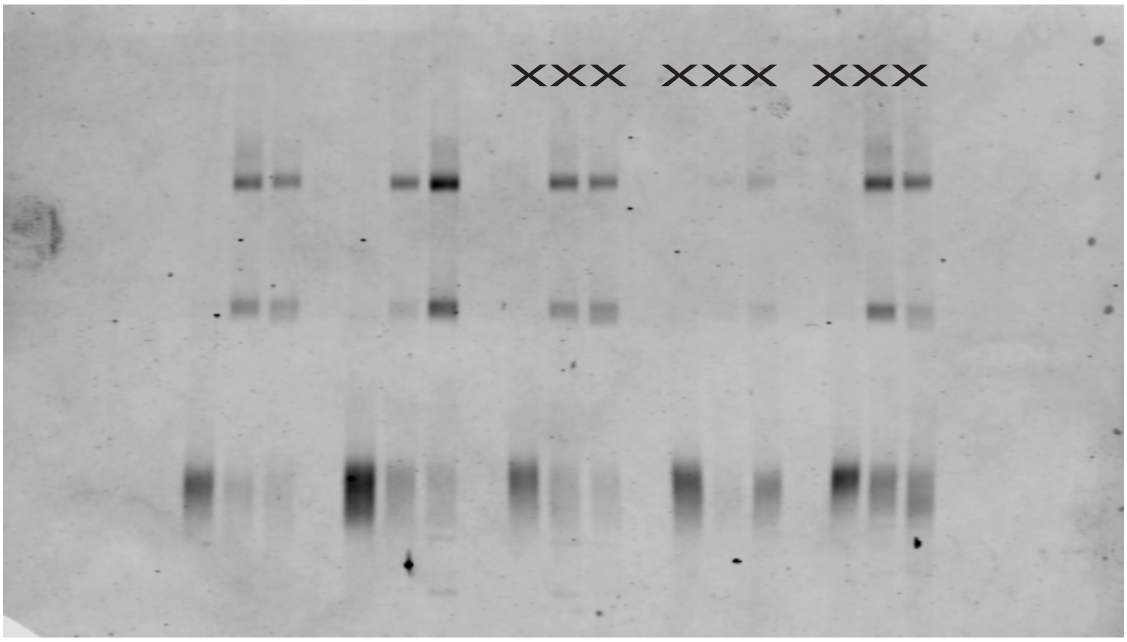

Genomic DNA digested with XhoI, separated on1% agarose gel, blotted and probed with a biotinylated RNA probe against the intergenic spacer region of the rDNA. Probe was detected with IRDye680 Streptavidin and scanned on a Licor Odyssey CLx

Sample order:

wt glucose log
wt glucose 24h
wt glucose 48h

wt galactose log
wt galactose 24h
wt galactose 48h

Used to generate Figure 5B

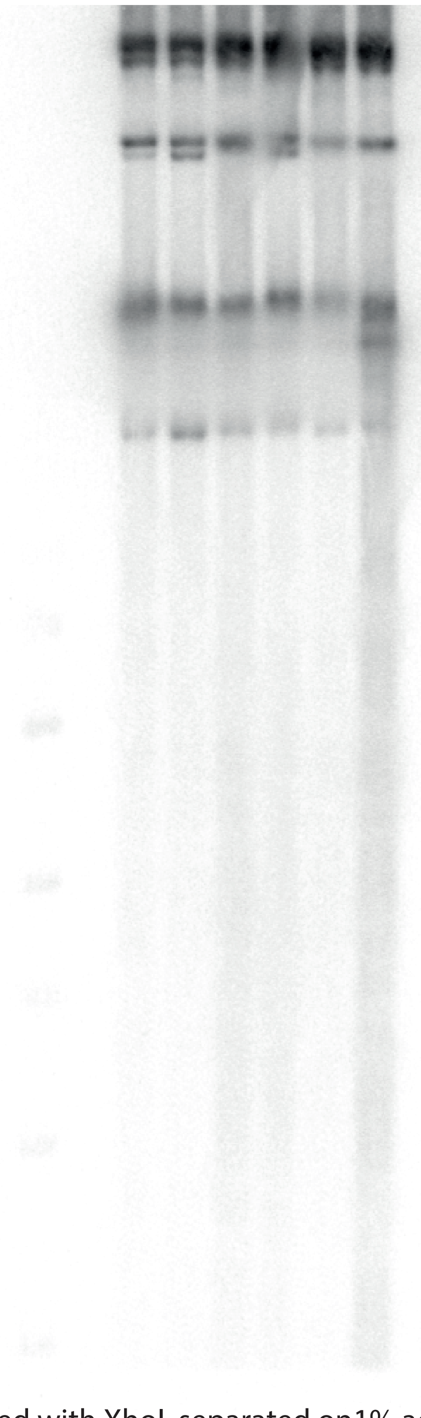

Genomic DNA digested with XhoI, separated on1% agarose gel, blotted and probed with a 32P random primed probe against the intergenic spacer region of the rDNA. Probe was detected with a Typhoon FLA3000 phosphorimager

Sample order:

wt glucose 48h (3 biological replicates)
wt galactose 48h (3 biological replicates)

Used to generate Figure S5C
