# Supplementary material for: Dietary change without caloric restriction maintains a youthful profile in ageing yeast
Source: PLoS Biol. 2023 Aug 29;21(8):e3002245. doi: 10.1371/journal.pbio.3002245 (PMC10464975; doi:10.1371/journal.pbio.3002245)
Supplement: Attachment — Submitted filename: Horkai et al response to reviewers.docx [file pbio.3002245.s012.docx]

# Horkai *et al.* Response to reviewer’s comments

Reviewer #1:

Replicative aging in yeast is characterized by a set of changes in cellular physiology, including the appearance of foci of the mitochondrial protein, Tom70, and global changes in transcription.  Many of these changes occur rather abruptly, late in the aging process, and these have been collectively termed the Senescence Entry Program (SEP).  In this manuscript, the authors show that growth in galactose rather than glucose suppresses the SEP, with senescence-associated changes happening either later, to a lesser extent, or both.  Despite this suppression of the cellular signs of aging, replicative lifespan is unaffected by the altered diet, thus the result could be summarized as an increase in "healthspan" without a concomitant increase in lifespan.  The authors go on to show that (1) suppression of the SEP depends on the ability to respire; (2) forcing respiration in glucose by overexpression of the transcription factor Hap4 also suppresses the SEP, although it does so only in a sub-population of cells; (3) cells that begin life growing in galactose maintain their youthful profile even after being shifted to glucose, but the converse is not true; and (4) levels of a fragment of chrXII that can be generated by replication problems at the ribosomal DNA, increase concurrently with the SEP.  One notable aspect of this work is that, although it has long been known that caloric restriction is beneficial with regard to replicative aging, the galactose diet used here is not calorically restricted compared to glucose.  The authors are careful to point out that, given that the change in diet does not increase lifespan, it is possible (though perhaps unlikely) that the changes in cellular physiology that they use to monitor cellular health are not the critical changes that drive aging.  This work is interesting, carefully executed, and clearly presented, and thus appropriate for publication in PLoS Biology.  Below, I offer comments and suggestions that I believe can improve the manuscript.

1.       	Assuming that the physiological changes associated with the SEP are detrimental to cellular health, it seems puzzling that cells grown in glucose don't have a shorter replicative lifespan than those grown in galactose.  The authors should comment on this in a more concrete fashion than "... showing that the mechanisms which limit lifespan are separable from those that cause senescence".  (In other words, if "healthy aging" doesn't prolong lifespan, and yeast don't really have "quality of life", then should it really be called "healthy aging"?)

We thought carefully about this point, and came to the conclusion that the only way in which we could define 'health' in budding yeast would be the maintenance of a rapid and uniform rate of division. We have added this definition to the Introduction as we agree that healthy ageing is not a term that is easily applied to yeast:

"We suggest that maintenance of a rapid uniform cell cycle provides the best definition of ageing health in budding yeast, and thereby ageing health must  decline dramatically at the SEP, which can be assayed using known SEP-associated markers."

We are aware that this is a somewhat subjective interpretation however, and therefore were careful to not describe our findings as 'healthy ageing' either in the manuscript title or the Results, restricting this only to the Discussion (and outlook sentence of the abstract where we interpret our findings in the context of the broader subject of ageing for which ageing health is a key concept.

2.       	The authors should note in the legend to Figure 2C that the gradations in gray shading in the background correspond to age.  Also in this figure, the gray panel on the right should be labeled "key to symbols", since, as it is, it looks like a stand-alone figure.

Done.

3.       	Figure 4 and its legend contain several errors (e.g. 2 Ds in the figure, no F, legend refers to things as heat maps when they aren't, etc.).

Thank you for pointing out these errors. Now corrected, along with equivalent errors in legend for Figure S4.

4.       	Mention how the ncRNAs at the rDNA correspond (or not) to c-pro and e-pro.  (I believe that IGS1-F and IGS1-R are e-pro, and IGS2-R is c-pro.)

We have annotated these in FIgure S5A and cross-referenced from the text.

5.       	Figure 5A is confusing.  Is the top row the forward strand and the bottom the reverse?  Also, the placement of the map of the intergenic spacer region in the middle of the two rows makes it look like the different columns are probing different regions of the two intergenic regions.

We see the reviewer's point here and have removed the schematic (which is included in S5A anyway) and organised the panels in a more standard form.

6.       	The following comment isn't plausible, since the ratio of intracellular NAD+ to NADH is much too high (60-700) for respiration to alter it enough to significantly alter Sir2 activity:  "The activity of the histone deacetylase Sir2 is promoted by respiration through the formation of NAD+ and clearance of nicotinamide (Lin et al., 2004), so it is reasonable to relate rDNA recombination events that form ChrXIIr to a lack of respiration."

We acknowledge that we have only referenced one of a controversial set of claims, and the reviewer's argument about the NAD+/NADH ratio is strong. The actual mechanism of Sir2 activation by respiration is not critical to the point we were making here, so we have changed this sentence as follows:

"The activity of the histone deacetylase Sir2 is promoted by respiration, though the underlying mechanism of this promotion remains unresolved (Anderson et al., 2003a; Anderson et al., 2003b; Lin et al., 2004; Lin et al., 2002), so it is reasonable to relate rDNA recombination events that form ChrXIIr to a lack of respiration."

7.       	"without restriction" in last sentence of abstract should be "without caloric restriction".

Fixed.

Reviewer #2:

In the manuscript the authors explore how different sugar sources impact aging phenotypes in budding yeast. They utilize the MEP, and metrics of SEP such as Tom70-GFP, Rpl13a-mCherry, RNA seq and bud scars which can be measure on thousands of cells with flow cytometry. They identify galactose as a sugar that prevents Tom70 accumulation with age and cells outcompete cells fed glucose (or several other sugars tested). Initial galactose feeding followed by later glucose has a similar effect. They speculate respiration levels form the foundation of the difference, and further explore this idea using cox9, gal80, snf4 deletions, and overexpression of Hap4 to alter respiration. The data seems very robust with many replicates and statistics. The authors propose two different aging trajectories. There is an advance but the generalizability of these findings to other species is not clear. The correlation between respiration, a change in Chr XII and SEP is interesting and significant but the causality and mechanisms involved are unclear.

We thank the reviewer for their insights. On the particular point of generalizability, we think that the changes in metabolism caused by a galactose diet could be translated to higher eukaryotes even if the galactose diet itself is yeast specific. We are investing a lot of effort in doing this at the moment and hope to have a publication ready next year.

 Comments for the authors consideration:

 1.       	Its somewhat difficult to compare the RNA seq profiles since the number of divisions are not always comparable at the timepoint collected.

We completely agree that this could be a problem, and took measures to minimise it in our work though we acknowledge that we did not make these steps very clear:

- A major reason we selected galactose for the initial analysis in Figure 1 was that preliminary experiments revealed cells on these two carbon sources reach the same average age after 24 and 48 hours of ageing as cells aged on glucose. We have added the following to the first paragraph of the Results: "...and ensured by bud scar counting that cells aged in glucose and galactose reached equivalent average replicative ages at 24 and 48 hours (Figure S1A)."
- Most other carbon sources similarly supported ageing at the same rate to glucose, with the exception of acetate that we noted was slow. We have added the following sentence: "Cells aged in fructose, sucrose, and raffinose/galactose attained equivalent replicative age at 24 and 48 hours to cells aged in glucose (Figure S1E), but cell division was slow in acetate, which prevents like-for-like comparison (Figure S1E), …"
- COX9 mutants die young, probably due to free radical production by Yno1 (PMID: 29912878), which we noted and considered in our interpretation of the data in Figure 3B/C).

In all other cases, the comparisons we make are between time-courses in which replicative ages were matched at each timepoint.

2.       	Its not clear to me why the Hap4 overexpression creates such heterogeneity in outcome. Is it integrated or on a plasmid that is heterogenous in retention/copy number? Why do some cells not retain expression? Do these cells still have the overexpression transgene construct?

This is a great question, and is very similar to the effects observed by Li et al of different ageing trajectories even within wild-type populations. The Hap4 overexpression construct is integrated, and both the old, healthy and young, unhealthy fractions show a similar *HAP4* mRNA level (Figure S4D). We have altered the relevant sentence in the results as follows: "..., median Tom70-GFP was significantly lower at 24 hours in MEP cells on glucose over expressing one chromosomal copy of HAP4 from a GPD promotor…".

As the *HAP4* mRNA does not differ, we think the most likely reason for the difference in Hap4 activity is a difference in iron homeostasis, which is known to follow different trajectories in ageing cells. We expand this idea in the Discussion .

3.       	Is the heterogeneity in the Hap4 experiment essential to the mixed outcome? Would high Tom70 outcompete low Tom70 that presumably have the same genotype (with the exception of the XIIr)?

We think this would depend on the environment. We have previously shown that as cells age on glucose and accumulate markers of ageing, they become less fit on glucose but more fit on other carbon sources (PMID: 28247585). We predict that the high Tom70-GFP subset would share this adaptable phenotype, and the split population is indicative of bet hedging in that yeast can tune the fraction of cells in the fit versus adaptable trajectories. This is a fascinating topic but one that we would prefer to address properly in a separate study on ageing and adaptation.

4.       	High Tom70 correlates with more gene dysregulation, but how well accepted is gene dysregulation as a metric of aging? The competition assay seems like a better indicator of health, but this experiment is not performed for any of the mutants.

Similar progressive gene expression dysregulation phenotypes during ageing in yeast have been very widely reported (Cruz et al., 2018; Hendrickson et al., 2018; Hu et al., 2014; Janssens et al., 2015; Kamei et al., 2014; Yiu et al., 2008), so we consider it a robust age associated phenotype even if the underlying mechanism is still debated. We used this heavily as a marker here because:

- Transcriptomic changes have been much more widely reported for yeast ageing than epigenetic, proteomic or metabolomic marks, so are definitely robust across labs and experimental systems
- Unlike the fluorescent markers we also employ, this is not affected by autofluorescence, photobleaching and other microscopy artefacts, and is not dependent on expression of a single gene.
- We have used competition assays frequently in yeast ageing work, and our experience is that they are much more variable and only reliable when performed by a very experienced experimenter, and only when the differences are very large. Therefore although we include competition assay data, we consider this much less robust than the RNA-seq data. Our RNA-seq protocol is routine and reproducible, such that three experimenters in our lab have obtained equivalent data for differences between ageing in glucose and galactose.

Overall, though not a classic marker of ageing in yeast, we consider gene expression changes to be a very robust metric that is orthogonal to the more common fluorescent markers which we also employ. It is also validated by the close correlation we observe between Tom70-GFP and gene expression slope values.

5.       	Are there differences in respiration levels between the different mutants? This could be informative for interpretation.

Yes, it is known that over-expression of *HAP4* increases respiration (PMID: 10788368), while *cox9* mutants are completely respiration incompetent and *snf4* mutants cannot grow on respiratory carbon sources (PMID: 6392017). We currently interpret the differences in senescence as being driven by respiration rates, at least for these mutants, and expand on this in the last section of the Discussion.

6.       	This manuscript helps to establish the correlation between the unusual chromosome structure and SEP, but Zylstra et al breaks this correlation with the strain that does not bear the unusual chromosome XIIr but still displays SEP.

This is a misunderstanding that we have attempted to address by better explanation in the Zylstra *et al* paper. Although the chromosomal translocation strain shown in Zylstra *et al* Figure 5F lacks the 590 kb region centromere distal to the rDNA, the ChrXIIr fragment still contains between 1 and ~180 rDNA repeats (up to 1.6Mb). In other words, the translocation just reduces the potential size range from 0.6-2.2 Mb to 0-1.6 Mb. If the presence of a large, non-centromeric chromosome fragment in the mother cell is associated with senescence then this is not prevented in the translocation strain. However, the fact that senescence still occurs in the translocation strain means that senescence is not caused by copy number amplification of one or more genes in the ChrXIIr region, which was a valid but incorrect hypothesis.

7.       	The authors use "non-ChrXIIr genes" as a control in several experiments, but it would be important to report also on only the rest of ChrXII as a control, since using the entire genome could easily wipe out non-euploid metrics.

We thank the reviewer for raising this issue. The actual control set was non-ChrXII genes, but was mislabelled on the figures and this has now been corrected. We have added a control for ChrXII left genes (from left telomere to rDNA), as the reviewer suggests, to Figure 5D and 5E and also to Figure 5C of Zylstra et al. There is a small and unconvincing rise with age in signal from this region in most assays, but in the fractionation experiment where ChrXIIr is much more prevalent we also see a clear increase in the ChrXII left region signal. We have noted this and interpreted it to mean that unresolved replication structures at mitosis can lead to retention of both Chromosome XII chromatids in the mother cell, though this occurs at a lower frequency than ChrXIIr formation. This fits with additional data we have added to Zylstra et al showing that some other chromosomes (I and VI) are also accumulated to a small extent in mother cells, in keeping with observations from Neurohr et al. This is now discussed in Zylstra *et al.*

8.       	The authors should comment on why respiration might protect against the SEP. Its counterintuitive since respiration is thought to generate ROS and DNA damage, and DNA damage is assumed to be problematic and a feature of aging.

Yes, increased respiration should increase ROS levels, but this cannot be a major problem for cells given that caloric restriction, which enhances lifespan and healthspan, also increases respiration (Anderson et al., 2003a; Lin et al., 2004; Lin et al., 2002; Schleit et al., 2013). We have added the following sentence to the relevant place in the Discussion: "This is somewhat counterintuitive, as increased respiration should increase levels of damaging reactive oxygen species (ROS), but low levels of ROS may actually be protective through the induction of hormesis (reviewed in (Ludovico and Burhans, 2014))."

9.       	Some mutations do extend lifespan although the manipulations in this manuscript do not. How do those mutants fit with the ideas presented?

We think that there are multiple ways to extend lifespan. Many mutants may extend the ageing timecourse along the trajectory normally observed in wild-type (thus extending the senescence period as well, as seems to be the case in *C. elegans* PMID: 26814965). However, given that one or more low senescence trajectories exist in yeast it is likely that other mutants favour these trajectories. The low senescence trajectory that we describe does not extend lifespan, but we do not know why cells on this trajectory become terminally arrested, and the low senescence trajectory described by the Hao lab does extend lifespan so the timing of terminal arrest does not appear to be an intrinsic part of the low senescence trajectory (PMID: 32675375).

This is a fascinating question, but one we will pursue in another manuscript as these suggestions are currently only speculation.

Figure 2, color shades for 48 and 72 hours are not distinguishable P3, "purified purified"

Fixed.

 Reviewer #3:

In the manuscript "Dietary change without caloric restriction maintains a youthful profile in ageing yeast" Horkai and Houseley compare aging trajectories of budding yeast cells grown on different carbon sources. Using purified populations of aged mother cells, they show that cells grown on galactose do not accumulate aggregated mitochondria, a hallmark of aging when cells are grown in glucose. Furthermore, the transcriptome of cells aged in galactose undergoes less changes during aging. Forced expression of the mitochondria biogenesis factor HAP4 in glucose partially induces the aging trajectory typically observed in galactose, suggesting that respirative growth slows cell senescence. Interestingly, they find that the aging trajectories are defined by the nutritional regime cells are grown at young age and cannot be reversed in old mother cells. Finally, the authors find that yeast populations that age more rapidly have increased expression from a chromosome fragment on the right arm of Chromosome XII (XIIr). In an accompanying paper from the same group they show that XIIr is tightly associated with cell senescence.

The manuscript presents a number of interesting observations, such as a suppression of age related phenotypes by a change of diet. While the authors point out that this nutrient regime does not constitute caloric restriction, cell growth in galactose is much (50%) slower than in glucose and thus the observation falls in line with many previous observations showing that slow growth slows replicative aging.

This is an important misunderstanding. Although bulk proliferation on galactose is slower than on glucose, the rate at which mother cells divide is not, so our findings cannot be attributed to growth rate differences. Data for this is shown in Figures 1B (WGA) and S1A (bud scar counting). This is a critical part of the underlying logic of our experiments and one that we did not sufficiently emphasise in the original manuscript. We now specifically make this point at the start of the Results:

"we used an optimised Mother Enrichment Program (MEP) protocol in which cells are biotin labelled then aged in rich media for 24 or 48 hours, fixed and mother cells purified (Figure 1A) (Cruz et al., 2018; Lindstrom and Gottschling, 2009), and ensured by bud scar counting that cells aged in glucose and galactose reached equivalent average replicative ages at 24 and 48 hours (Figure S1A)."

Accumulation of a fragment of chromosome XII has been reported before, but the tight association of ChrXIIr accumulation with cell senescence is interesting and suggests a direct link between these two processes. The observation that respiration, and in particular forced expression of HAP4, changes the aging trajectory has been made previously as acknowledged by the authors. While I think these observations are of interest to the readers of PLOS Biology, I have a number of concerns with the manuscript. In particular the interpretation of the transcriptome data seems complicated by factors that are not controlled for as outlined below. The first point applies to both manuscripts and parts of it are a direct copy.

1)       	My main concern is the interpretation of the transcriptome data. The indicated age dependent decrease in ribosome biogenesis genes is reminiscent of activation of the environmental stress response (ESR), which is a hallmark of aging, slow growth and permanent cell cycle arrest (PMID: 22498653, PMID: 17105650, PMID: 30739799). A terminal cell cycle arrest is entered at the end of replicative life irrespective of the reason of why and how fast cells aged. The fraction of terminally arrested cells in each population of purified aged mother cells could thus dominate the transcriptome profile. This is for example evident in the Hap4 overexpression data in Figure 4: Cells that have undergone only few divisions (low WGA population) have likely been arrested for many hours and display a strong change in gene expression on the MA plots (ESR?) while cells that are still cycling (high WGA population) have not been arrested and show a weak gene expression change. The authors need to control their transcriptome data for this effect by A) determining division times and the fraction terminally arrested cells in the aged populations and B) comparing what part of the gene expression change is driven by activation of an ESR, for example by comparing to published data sets of slow growing or arrested cells.

We agree with much of this argument, but our data shows that age-linked gene expression changes cannot be attributed to terminal arrest since at 48 hours similar numbers of cells are terminally arrested in glucose and galactose (if anything more in galactose) (Figure 1D), yet we only see a strong gene expression effect in cells aged in glucose.

We also agree with the reviewer that the gene expression change is very similar to the ESR. This may represent active responses to stress or slow growth, or a combination of both (PMID: 18753408, PMID: 24952590). We know that the cell cycle slows dramatically after the SEP, so in retrospect it is not too surprising that the SEP is associated with an ESR-like gene expression signature.  In Zylstra *et al.* we have now included a substantial analysis addressing the contribution of the ESR and growth rate to the SEP.

We suspect the difference between glucose and galactose is that whatever causes terminal arrest of cells on galactose happens quickly and the cells do not have time to adjust gene expression for the new growth rate, whereas in glucose the majority of cells have an extended period of slow growth prior to complete arrest, during which gene expression is adjusted.

We have added a paragraph to the Discussion on this point.

If the gene expression profile in aged populations is a consequence of slow growth or prolonged cell cycle arrests, it changes the interpretation of several figures. For example:

 a.       	Figure 2A, C: PC1 (aging) component correlates with slow growth rate (log phase cells in galactose grow 50% slower than cells grown in glucose, cells grown in acetate grow even slower and already show an "aged (PC1)" trascriptome). As a consequence, cells that already grow slowly when young will show a smaller shift in PC1 as they age (this includes also the acetate data) and a flatter slope in the MA plots in 2A. This is different from the interpretation that growth in galactose prevents age induced gene expression changes.

We selected galactose versus glucose as the primary comparison for this work because, although bulk proliferation in culture is indeed slower on galactose than glucose, the average mother cell division time is equivalent for glucose and galactose. Evidence for this:

- Bud scar counts are equivalent after 24 and 48 hours on glucose and galactose - see Figure S1A
- WGA staining of bud scars is equivalent at 24 and 48 hours on glucose and galactose across many experiments (Figure 1C)
- Manual measurements of cell cycle time during ageing by microdissection show similar times for glucose and galactose - see Figure 2C in PMID: 28247585.

The slow average cell cycle time in bulk galactose cultures relative to glucose must therefore be due to newborn daughter cells (which form 50% of bulk populations and take longer to reach the required size for division than mother cells). But, the reviewer is absolutely correct that the measurements of slope are very dependent on the non-aged reference, and our use of log phase cells as a reference could be a problem because it contains 50% new-born cells.

To address this concern, we have added MA plots comparing 48 hour aged cells to 7.5 hour old cells which are young (average 5 divisions) but free of slow growing new-born cells (Figure S2A). These plots are equivalent to plots comparing 48 hour to log phase cells, showing that the gene expression effect we observe is not an artefact of using log phase reference samples. The only notable difference is that there is less change in ribosomal protein genes with age when log phase cells are removed, which doesn't affect the point we are making but does reduce the slopes measured by linear regression for both carbon sources.

We have added the following sentence describing this experiment:

"Equivalent  results were obtained comparing mothers harvested after 7.5 and 48 hours (Figure S2A), showing that the observed differences between glucose and galactose are not driven by the large fraction of new-born daughter cells in the log-phase samples."

b.       	Figure 4C-D: Low WGA cells have undergone fewer divisions than high WGA strains. These low WGA strains have therefore probably been in a terminal cell cycle block for many hours. This data strongly suggests that the slope in the MA plot is a consequence of terminal cell cycle blocks. Because low WGA cells also loose the Hap4 induced gene expression signature, the authors conclude from this data that HAP4 overexpression and respiration are protecting cells from entering senescence. An alternative interpretation is that HAP4 over-expression leads to premature permanent cell cycle exit in about 50% of cells (=low WGA cells). To distinguish between these possibilities, the Tom70-GFP vs WGA plot (4B, right) should be shown for both wild type and PGPD:HAP4 cells.

This is a good point. We have generated the equivalent graph for the matched wild type (Figure S4B), and observe that the distribution looks very similar to the Low WGA High Tom70 population in Pgpd-HAP4. Therefore HAP4 expression is indeed protective to ~50% of cells. We have added the following sentence for this graph: "

An equivalent plot of wildtype cells shows that the high WGA, low Tom70-GFP population is largely absent (Figure S4B)."

2)       	Cells grown on YP galactose grow roughly 50% slower than on YP glucose. It is therefore surprising that these cells age at the same rate (as suggested by the WGA staining in Figure 1B). The authors should address this issue more carefully, for example with manual bud scar counting at the different timepoints or by performing pedigree analysis.

Yes, as discussed in answer to point 1A above, we think that this difference between population proliferation rate and rate of replicative ageing arises because newborn buds are slower to reach maturity in galactose. The requested data including bud scar counting and WGA intensity is given in Figure S1A.

3)       	The fact that cells aged on galactose maintain fitness (Tom70-GFP, fitness assay) even though they age at the same rate (viability, WGA staining) as cells aged on glucose is surprising and somewhat contradictory. It would be interesting to repeat the competition assay after 48 hours of aging where the difference between the two conditions (SEP marker Tom70-GFP) seems to be even more exaggerated. Ideally this experiment would be combined with manual bud scar counting, which is more accurate than WGA intensity, especially if cell size differs between conditions.

This is a frustratingly difficult experiment, hence our inclusion of fitness assays only at 24 hours. Aged cells are quite delicate and often lose viability on handling, media changes, etc, and this seems to affect galactose aged cells more than glucose aged cells. The result is that we can obtain viability data based on colony formation which requires absolutely minimal handling, but the cells often die during or just after setting up competition fitness assays. This is not a problem for the huge differences in fitness we measured in our Frenk et al study, but means that reproducibility is poor for more subtle differences such as glucose versus galactose.

To provide a measure of fitness in these very old cells, we have performed very gentle live cell purifications at 48 hours in which the cells are never free of media. We then placed individual mother cells at defined sites on YPD plates using a micromanipulator and measured colony size after 24 hours. Remarkably, almost all viable colonies arising from 48 hour cells aged on galactose were equivalent size to colonies formed by young cells, which demonstrates that there is minimal loss of fitness with age, whereas all colonies formed from 48h cells aged on glucose were smaller, with many cells not dividing at all in the first 24 hours and only giving rise to colonies over the next few days. This shows that cells aged on glucose whilst still viable are dividing extremely slowly. We note that this approach could not be combined with bud scar counting as bud scars are not visible using the micromanipulator microscope.

We have included this data as Figure 1F.

4)       	The key definition of the SEP is a lengthening of cell division time. Tom70-GFP is one of several markers (as is nucleolar enlargement). The use of Tom70-GFP as senescence marker is especially problematic in strains that carry mutations that affect mitochondria biogenesis (snf4∆, PGPDHAP4). As alternative, the authors should measure cell cycle durations of aged mother cells aged in Glc or Gal to determine whether or not they have passed SEP (since increase in cell cycle duration is the key defining feature of the SEP).

We completely agree with this, but from our point of view cell cycle duration data would only be useful if performed under equivalent conditions to the other experiments in our study; this is remarkably difficult and we have not found a good solution. We formed a collaboration with the Charvin lab to perform microfluidics analysis in glucose and galactose but the experimental conditions we use are incompatible with microfluidics. The devices can only accommodate haploid cells, whereas our study was performed in diploids, and require synthetic media whereas our study used rich media. Even more problematically, the effects of caloric restriction are not well replicated on microfluidic devices, and the long lived low senescence fraction of wild type populations reported by the Hao lab are not seen in devices used by other labs. As we noted in the discussion, it is therefore currently extremely difficult to validate our low senescence results in microfluidic devices. We are pursuing this with the Charvin lab but solving these problems is beyond the scope of the current study.

We note however, that we have added nucleolar size data to our revised manuscript (Figure S5C): this increases in glucose not galactose in accordance with our Tom70-GFP and Vph1-mCherry data. Also, the fitness experiment discussed in point 3 above clearly shows that cells aged in glucose are cycling more slowly than those aged on galactose.

5)       	The WGA staining of snf4∆ mutant cells is high in both log and aged cells. Does that mean that snf4∆ mutant cells are born old or that WGA intensity is not in all cases a good measure for replicative age? This is not discussed in the text.

 We thank the reviewer for pointing this out - we had inverted the columns for wt 24h and snf4 log in figure preparation. The corrected graph shows no anomalies in WGA level.

6)       	Figure 5B: The ncRNA expression at the 48h timepoint is similar between Glc and Gal and so are the levels of ERCs. The more interesting timepoint in this regard would be the 24h timepoint. From the ncRNA expression I would predict that ERCs accumulate more slowly in Galactose - which would be consistent with the hypothesis that slow growth affects ERC formation and through that aging.

We have replaced Figure 5B with a southern blot for ERCs on glucose and galactose across log, 24 and 48h, and moved the blot showing quantification at 48h to Figure S5C. The accumulation of ERCs follows the ncRNA dynamics in Figure 5A as expected. The reviewer is correct that ERCs accumulate more slowly in galactose, but it remains very hard in a model of ERC-induced senescence to reconcile the black-and-white difference in senescence markers at 48 hours with the fact that the ERC levels are identical.

Please note that the new southern blot looks qualitatively different to the previous ones here and in Zylstra et al. This results from our recent move to using fluorescence instead of ^32^P for labelling southern blot probes.

 Reviewer #4:

In this manuscript, Horkai and Houseley report that growth on galactose media alleviates the impact of aging on yeast. They further report that the reduced impact is preserved even if the media is changed from galactose to glucose later in life. As their main aging markers, the authors use Tom70-GFP fluorescence as an SEP marker as well as gene expression levels/dysregulation measured by RNAseq.

While the idea of this manuscript is interesting, I have major concerns about the selection of aging markers, interpretation of the data and the conclusions reached.

1. To convincingly prove the "healthspan" impact of galactose over glucose AND the early galactose exposure's preserving its effect later in life, the authors must use more well-established aging markers and show that the markers are reduced in amount/intensity and/or onset for appearance in cells.

This is an important point and to strengthen findings with Tom70-GFP, we include two other markers drawn from separate studies:

Firstly, vacuolar structure and pH changes during ageing in glucose are thought to underlie mitochondrial defects (PMID: 23172144). In this publication, both area and intensity of the vacuolar marker Vph1-mCherry are shown to increase with age in glucose. We find that age-linked increase in intensity of Vph1-mCherry is robustly suppressed during ageing on galactose, and the increase in vacuole size is lessened  (Figure S1B).

Related text: "Vph1-mCherry intensity and area increased substantially with age in glucose, showing that the Tom70-GFP phenotype is not an artefact of a single protein or fluorescent tag, but Vph1-mCherry intensity was almost unchanged with age in galactose (Figure S1B, left), while the increase in vacuole size was significantly lessened (Figure S1B, right)."

Secondly, using the RNA pol I marker Rpa190-GFP we have assessed nucleolar size and intensity, both of which are reported to increase on entry to senescence in glucose in microfluidic systems (PMID: 31291577). We observe an increase in nucleolar size with age in glucose, but this does not occur in galactose. This data is included in Figure S5C as it is very relevant to the measurements of ERC abundance. We note that Rpa190-GFP intensity does not robustly increase with age in glucose as Morlot et al observed, but dramatically declines by 48 hours; this behaviour exactly mirrors the behaviour of the ribosomal marker Rpl13a that is shown in Figure 1B, and we think this represents a down-regulation of ribosome synthesis in post-SEP cells that are growing slowly and require less ribosomes. Again, cells ageing in galactose do not show dramatic changes in Rpa190-GFP signal, so the Rpa190 data also reinforces our argument that the SEP is suppressed during ageing in galactose.

Related text: "It has been proposed that ERC accumulation is the cause of the nucleolar enlargement which accompanies ageing beginning at the SEP (Morlot et al., 2019; Sinclair et al., 1997) so we measured nucleolar size using the RNA polymerase I reporter Rpa190-GFP. In keeping with other markers of the SEP, nucleolar size increased with age on glucose but not on galactose, reinforcing our conclusion that galactose-aged cells do not become senescent (Figure S5C, top). This observation is surprising since ERC levels in 48 hour-aged cells are not different between glucose and galactose; combined with the observation in Zylstra et. al. that nucleolar enlargement also occurs in cells lacking ERCs, we conclude that ERC accumulation cannot be the cause of nucleolar enlargement. Rpa190-GFP signal intensity also did not correlate to nucleolar area, and recapitulated behaviour of the ribosomal protein Rpl13a-mCherry marker (compare Figure S5C bottom panel to Figure 1B middle panel). On glucose, Rpa190 intensity increases slightly at 24 hours but then decreases to below log phase levels in most cells, presumably because cells divide very slowly after the SEP and have a lower demand for ribosomes, whereas on galactose Rpa190 levels change little with age, reflecting a steady requirement for ribosomes."

We therefore have a complementary set of markers reported to change with age and/or senescence in different publications addressing yeast ageing, all of which are suppressed when cells age in galactose: Tom70-GFP (characterised in PMID: 24332850 and PMID: 32675375), Vph1-mCherry (PMID: 23172144), Rpa190-GFP (PMID: 31291577). We consider this to strongly support our conclusion that cells ageing in galactose (at least diploid cells ageing in culture in galactose) do not enter senescence during ageing.

2. Unless I missed it, the authors don't directly show the budding interval comparisons between galactose and glucose environments in diploid cells. A microfluidic device could readily provide that data. Without this growth rate information, 24hr and 48 hr time points could mean or correspond to different age points in each media or when the two media were swapped.

Yes - this is very important. We provide data in Figure S1A showing that bud scar counts are equivalent for glucose and galactose aged cells at 24 and 48 hour time points. This means that the cells are of the same age at the given time points in the different conditions. We have also previously reported that budding intervals of aged cells in glucose and galactose are similar based on micro dissection (PMID: 28247585).

This equivalence of cell division rate in mother cells was the primary reason we pursued galactose as the non-glucose carbon source for this study.

With regard to the microfluidics device, we formed a collaboration with the Charvin lab to perform microfluidics analysis in glucose and galactose but the experimental conditions we use are incompatible with microfluidics. The devices can only accommodate haploid cells, whereas our study was performed in diploids, and require synthetic media whereas our study used rich media. Even more problematically, the effects of caloric restriction are not well replicated on microfluidic devices, and the long lived low senescence fraction of wild type populations reported by the Hao lab are not seen in devices used by other labs. As we noted in the discussion, it is therefore currently extremely difficult to validate our low senescence results in microfluidic devices. We are pursuing this with the Charvin lab but solving these problems is beyond the scope of the current study.

3. The claimed gene expression changes/dysregulation data is not very convincing or informative. The whole transcriptomic profiles are reduced to slopes of lines. The analyses should ideally be performed in a gene-specific manner across the time points. The bulk RNAseq data plotted/analyzed at the population level is not very informative. The conclusions are drawn from the changes in the slope of the linear fitted line, and this is not very convincing.

In our original analysis we also looked at genes individually based on differential expression across conditions and timepoints. The respiration effect was the primary outcome of this analysis (Figure 3A). We were hoping to find more clusters of genes that behaved differently between the conditions, but actually we just observed the whole set of differentially expressed genes moving in the same directions but to a greater or lesser extent in the different media conditions. The slope value of the gross population captures this difference well, hence our use of slope calculated by linear regression as a primary metric.

However, on reflection, we do accept the reviewer's point that this approach is overly reductive and does not convey the behaviour of individual genes. We have therefore extracted the sets of genes that are significantly differentially expressed with age in glucose, and displayed the behaviour of these genes across age in the other conditions (Figures 2D, S6B).

One complication in these analyses is that the change in the baseline slope of the population renders comparison between young and old datasets by DEseq2 (and similar packages) invalid. To obtain datasets that can be used for differential expression analysis, we applied a quantile normalisation that essentially removes the slope differences that we measured throughout the original manuscript. This normalisation is visible on the new MA plots as the data is uniformly distributed around the X-axis (eg: Figure 2D).

Relevant text: "It is clear from the PCA that PC1 dominates the datasets, but this is tightly associated with the distortion of the whole gene expression profile that we represent in the slope value. This distillation of the whole transcriptome change to a single value is very reductive and we asked whether subsets of genes are acting differently to the bulk dataset during ageing in different carbon sources. We used quantile normalisation to remove the overall profile difference represented by the slope from the datasets, extracted genes differentially expressed during ageing in glucose then highlighted these genes on  MA plots for glucose, galactose and fructose ageing (Figure 2D). While the normalisation is effective at removing the baseline distortion, it is still obvious that gene expression changes much less during ageing on galactose compared to glucose or fructose. No subsets of genes change in behaviour, and we simply observe that the gene expression changes that occur during ageing in glucose also occur in galactose, just to a much lesser extent."

Although we were aiming to find defined gene expression signatures of ageing health in this dataset, we actually observe very little evidence of high effect genes; rather the effects of ageing on gene expression seem primarily to involve global redistribution of mRNA abundance.

4. The authors claim/state in key positions of the manuscript (e.g. abstract) that the yeast lifespan is similar between glucose and galactose. This is not true based on the Liu et al paper cited by the authors. Since galactose lowered yeast lifespan in that paper using a direct single-cell lifespan assay, the concerns raised about this manuscript above (healthspan-related conclusions reached by non-standard aging marker and bulk RNA data analyzed at the slope-of-the-population level) becomes even more important/valid.

While we understand the reviewer's concern - lifespan was 17% lower in galactose as measured by Liu et al, that was not our point. Our concern was that the suppression of ageing phenotypes at equivalent ages by galactose growth could arise if the whole life course is massively extended, such that ageing phenotypes occur for the same proportion of lifespan but emerge at a higher absolute age. Given that dietary restriction, the classic intervention for improving ageing health, extends lifespan significantly in *S. cerevisiae*, we considered it important that the lifespan was not longer. We have altered the relevant wording to read 'similar or shorter' or 'not longer' throughout the manuscript, but this does not alter our conclusions. If the cells have a similar (as we measure) or shorter (as Liu et al measure) lifespan then the suppression of ageing phenotypes at fixed time points is not simply due to extension of lifespan. We have added data to address concerns about markers and gene expression phenotypes as discussed above.

5. Taken together, the current state of the manuscript does not support several of the conclusions written. As an example: "Given that lifespan is not different between glucose and galactose, this means that gene expression dysregulation is an aspect of ageing that is not intrinsically associated with increasing replicative age or lifespan."

This bold statement is simply not true.

We take the reviewer's point that this should read 'little different' and we have changed this sentence appropriately. We do not detect a significant difference in lifespan, but our data is not inconsistent with the slightly shorter lifespan in galactose measured by Liu et al as their method for lifespan determination is definitely more accurate than the method we need to use for MEP cultures.

However, the point we are making here is still correct: age linked gene expression changes that occur in glucose do not occur in galactose aged-cells of equivalent replicative age, and we see no correlation to lifespan: galactose aged cells show far less gene expression dysregulation than slightly longer lived glucose aged cells, but in the Pgpd-HAP4 experiment it is the younger cells that enter replicative senescence early which show strong gene expression changes. These diametrically opposite observations mean that gene expression changes cannot be intrinsically associated with lifespan.
