# Supplementary material for: Dietary change without caloric restriction maintains a youthful profile in ageing yeast
Source: PLoS Biol. 2023 Aug 29;21(8):e3002245. doi: 10.1371/journal.pbio.3002245 (PMC10464975; doi:10.1371/journal.pbio.3002245)
Supplement: Attachment — Submitted filename: Horkai et al second response to reviewers.docx [file pbio.3002245.s013.docx]

**Horkai *et al.* response to reviewers’ comments**

We thank the reviewers and editors for their time and thoughtful comments.

Reviewer #3:

 The authors have shown a good effort and have mostly addressed my concerns. I have raised my concerns about the role of the XIIr in the accompanying manuscript and will not repeat those here. One remaining point the authors should address is the role of different growth rates in driving the gene expression patterns:

My concern about growth rate and aging was partially addressed by showing that mother cells divide at the same rate using bud scar labeling. However, the overall slower population doubling time in poor carbon sources stems from a decreased rate of biomass synthesis, which is completely directed towards daughters during S/G2/M. This results in small daughter cells that take longer in G1 while mother cells apparently can progress through cell division at the same speed as mother cells grown in glucose. Nevertheless, the slow biomass accumulation is characterized by slow a growth transcriptional signature, which reflects reduced ribosome biogenesis and translation rates. Hence my comment that the repression of aging phenotypes in galactose is in line with previous reports about conditions that reduce cell growth delaying aging.

This is an interesting point that we had not considered, and we agree that the amount of biomass produced as opposed to the cell cycle rate or cell size of the mother may actually be a critical factor. We have added the following paragraph to the Discussion to encapsulate this:

"Longer lifespan and improved health-span are broadly associated with reduced biosynthetic activity (i.e.: slower growth), including mutants affecting TOR signalling, PKA signalling, ribosome synthesis, and growth under dietary restriction (Chiocchetti et al., 2007; Kaeberlein et al., 2005; Lin et al., 2000). Although mother cells divide at similar rates on glucose and galactose, population growth is slower in galactose implying that the daughter cells begin life with less biomass resulting in an extended growth period before the first cell cycle. For the mother cell this would mean less biosynthesis per generation, and the observed improvement in health-span is therefore consistent with the idea that reduced biosynthetic activity is beneficial for ageing fitness. Our media shift experiment shows that early life defines ageing trajectory, and we speculate that it may be this difference in growth of new-born daughters prior to the first cell division that defines the ageing trajectory followed for the rest of life."

Also, my comment about the MA plots was referring to the fact that cells that already start with a slow growth transcriptional signature will have a smaller change in PC1, which is presumably driving the slope in the MA plots.

 Yes, this definitely contributes to the difference in slope. We have added the following paragraph to the Results to make this clear:

"Ribosome synthesis is tightly coupled to growth rate (Waldron and Lacroute, 1975), suggesting that growth rate is a major contributor to PC1; although we observe that mother cell cycle rate is equivalent between glucose and galactose, population doubling time in galactose is longer indicating slower growth of new-born daughters. Indeed, log phase samples on glucose are located more towards the high ribosome synthesis/translation end of PC1 than log phase cells on galactose, reflecting faster population growth. Nonetheless, highly aged cells on glucose are located further towards the low ribosome synthesis/translation end of PC1 than equivalently aged cells on galactose, consistent with the lower Rpl13a-mCherry signal and the lower fitness of glucose-aged cells compared to galactose (Figures 1C and 1F). As shown in the accompanying manuscript (Zylstra *et al.*), the slope change on MA plots during ageing arises from slowing of growth, so the difference in slope between glucose and galactose aged cells reflects both the faster growth when young and the slower growth when old of cells on glucose compared to cells on galactose."

Reviewer #4:

I wish I could be more positive, but unfortunately, I am not currently satisfied with some of the responses given by the authors to the major points I had raised. I totally agree with reviewer 3 that "The key definition of the SEP is a lengthening of cell division time". The authors should have measured cell cycle durations of mother cells aged in Glucose or Galactose to determine whether or not they have passed SEP. Liu et al already has the longitudinal single-cell aging data (even though in haploids), the further analysis of which should have direct answers to several of the SEP-related questions posed by the reviewers and currently unaddressed by the authors. When the galactose lowers yeast lifespan by 17% (which is not "slight" as the authors call it), and when the direct/logical reporter of SEP (lengthening of cell division time) is not used during the course of aging, many of the conclusions of this manuscript are still up in the air.

For example, the first paragraph of the Discussion section reads "Here we demonstrate that substitution of galactose for glucose suppresses cell intrinsic processes that lead to a spectrum of seemingly pathological changes during yeast replicative ageing. This does not extend lifespan, showing that the mechanisms which limit lifespan are separable from those that cause senescence.""  This actually limits lifespan and the overall results are not interpreted well in the presence of lifespan reduction in galactose. At the least, this paragraph suggests that there is no lifespan consequence of growth on galactose, when the Liu paper showed that there is. In other words, there are several places in the manuscript that does not explain the counterintuitive results in the presence of 17% lifespan reduction by galactose. On top of that, the SEP is not measured using the metric of the lengthening of cell division time. Taken together, this manuscript needs further work to be complete/definitive.

If youthful profile or healthy aging or healthspan in yeast is defined by the ability of cells to maintain "clock like" low cell-cycle durations generation-after-generation (and this is a very good definition, perhaps the only one), then it is a practical requirement to measure generation-specific cell-cycle durations across media conditions. This manuscript is aiming to address galactose's being better than glucose in facilitating youthful profile. Therefore, anything shorter than measuring cell-cycle durations and SEPs in terms of altered division times would fall short for a strong paper.

As a final note (that again emerges from the Liu et al paper), it looks like the anti-correlations between cell-cycle durations, their (small) variations across lifespan (as a proxy for cells not as we entering senescence) and the lifespan itself have been studied at the correlation level by the Acar lab. Going to the single-cell cell-cycle trajectories, grouping "silent" and "noisy" trajectories and looking at their final lifespan could provide further insights into some of these questions.

While we understand the reviewer's frustrations here, we discussed in the previous rebuttal the effects of galactose we observe in diploids in batch culture in rich media do not appear to be recapitulated effectively by haploids in microfluidics systems in synthetic media, and so the required experiment is not technically possible. As we note in the Discussion, this is the same problem observed for caloric restriction, which also has a very weak effect in microfluidic systems. Neither result is wrong, it is simply that ageing as a complex process is affected differently by dietary manipulations under experimental conditions. Our point was not to demonstrate that galactose is a 'cure-all' for ageing, but rather that within a given system it is possible to find unrestricted diets that effectively move cells to a healthy ageing trajectory. The focus for us now will be to find genetic interventions that provide equivalent healthspan benefits irrespective of diet, and we hope that these will be more tolerant across experimental systems.

We did not mean to understate the importance of a 17% reduction in lifespan, and have removed the word 'slight', but our point remains valid that on galactose cells are reaching very advanced age, pretty much the limit of replicative lifespan, without showing any of the molecular markers of pathology widely observed on glucose and without losing fitness.
